# Complete Genome Sequence of a Novel Hafnia alvei Bacteriophage

**DOI:** 10.1128/mra.00049-22

**Published:** 2022-02-28

**Authors:** Alexander Bullen, Alex Grahe, Penka Kassolis, David R. Singleton

**Affiliations:** a York College of Pennsylvania, York, Pennsylvania, USA; Queens College CUNY

## Abstract

A bacteriophage that is able to infect Hafnia alvei and can also infect two other hosts, Klebsiella pneumoniae and Salmonella enterica, was isolated from wastewater. The complete genome sequence was determined by long-read PacBio sequencing. Based on sequence similarity, the bacteriophage is assigned to the viral genus *Kolesnikvirus* within the family *Myoviridae*.

## ANNOUNCEMENT

Hafnia alvei is the type species of the genus *Hafnia* and is a common member of the human gastrointestinal microbiota. It has not historically been seen as a significant pathogen; however, it is increasingly being recognized as a cause of infection in various body sites, particularly in cases of patients with comorbidities (1). A single report in the literature describes a H. alvei bacteriophage (2) but does not characterize it molecularly.

Bacteriophage vB_HalM_SPARTY (phage SPARTY) was recovered from untreated wastewater in York, Pennsylvania (40.01394142344336, −76.80265132387892). H. alvei strain 04414 (ATCC 9760) was used as the host to isolate the phage using the tryptic soy broth culture enrichment method (3). The isolated phage formed small (2-mm-diameter) clear plaques on soft agar overlays with overnight growth at 25°C; however, significant plaque turbidity was seen with growth at a higher temperature (37°C). Plaques were also observed using Klebsiella pneumoniae (ATCC 9997) and Salmonella enterica subsp. *enterica* (ATCC 14028) as the hosts, although zones of lysis were not as prominent as with H. alvei.

The phage was amplified with the plate lysis method (4), and genomic DNA was prepared using a Wizard DNA clean-up system (Promega Inc., Madison, WI). PacBio Sequel next-generation sequencing (NGS) was performed by Genewiz, Inc. (South Plainfield, NJ). Purified DNA was fragmented and end repaired, followed by adaptor ligation for DNA sequencing reactions, using the SMRTbell Express template preparation kit v2.0 (PacBio, Menlo Park, CA). A total of 395,155 polymerase reads, with a polymerase *N*_50_ value of 23,237 bp, were obtained from Genewiz as fastq sequence files.

Canu v2.2 (5) was used to assess DNA sequence read quality, and the reads were then *de novo* assembled into an initial contig of 89,029 bp. Program defaults were used for assembly and analysis unless noted otherwise. Genome termini were identified using PhageTerm v1.0.12 (6) (at https://Galaxy.pasteur.fr), which reoriented the sequence to a final consensus of 85,494 bp (GC content, 43.7%). PhageTerm suggested that this bacteriophage is a direct terminal repeat (DTR) class phage similar to phage T7, with 439-bp terminal repeats.

The genome sequence was imported into DNA Master v5.23.6 (http://cobamide2.bio.pitt.edu) for annotation. Potential tRNA genes were identified with ARAGORN v1.2.41 (7). Potential protein-coding sequences were identified with GeneMarkS v4.28 (8), GLIMMER v3.02b (9), and Prokka v1.14.5 (10) and compared for consistently identified open reading frames. BLASTp v2.12 (11) was used to tentatively assign protein functions. A total of 116 total potential protein-coding sequences and 27 tRNA genes were identified. Of the 116 potential protein-coding sequences, 35 demonstrated similarity to known phage proteins, and 81 had domain homology to proteins with no clear function or were hypothetical in nature.

Whole-genome alignment using BLASTn v2.12 (11) identified phages vB_SenM_SB18 (GenBank accession number MK759884.1) (99.1% sequence identity to phage SPARTY, with host Salmonella enterica), SunLIRen (GenBank accession number MH426725.1) (98.1% sequence identity, with host Erwinia amylovora), and phi63_307 (GenBank accession number MG589384.1) (97.7% sequence identity, with host Chronobacter sakazakii). The phylogenetic relationship of these phages was determined by whole-genome comparison using GRAViTy (12) (http://gravity.cvr.gla.ac.uk), placing phage SPARTY within the family *Myoviridae* in the viral genus *Kolesnikvirus*.

### Data availability.

The finished vB_HalM_SPARTY genome is available under GenBank accession number OK483342. NGS reads in fastq format are available under Sequence Read Archive (SRA) accession number SRR17309726, with BioSample accession number SAMN24299146 and BioProject accession number PRJNA791628.

## References

[1] Begbey A, Guppy JH, Mohan C, Webster S. 2020. *Hafnia alvei* pneumonia: a rare cause of infection in the multimorbid or immunocompromised. BMJ Case Rep 13:e237061. doi:10.1136/bcr-2020-237061. PMC7780556 33384344

[2] Guinee PA, Valkenburg JJ. 1968. Diagnostic value of a *Hafnia*-specific bacteriophage. J Bacteriol 96:564. doi:10.1128/jb.96.2.564-.1968. 5674062 PMC252333

[3] Twest R, Kropinski AM. 2009. Bacteriophage enrichment from water and soil, p 15–21. *In* Clokie MRJ, Kropinski AM (ed), Bacteriophages. Humana Press, Totowa, NJ. 10.1007/978-1-60327-164-6_2 19066806

[4] Ausubel FM, Brent R, Kingston RE, Moore DD, Seidman JG, Smith JA, Struhl K. 1992. Short protocols in molecular biology: a compendium of methods from Current Protocols in Molecular Biology. Cold Spring Harbor Laboratory Press, Cold Spring Harbor, NY.

[5] Koren S, Walenz BP, Berlin K, Miller JR, Bergman NH, Phillippy AM. 2017. Canu: scalable and accurate long-read assembly via adaptive *k*-mer weighting and repeat separation. Genome Res 27:722–736. doi:10.1101/gr.215087.116. 28298431 PMC5411767

[6] Garneau JR, Depardieu F, Fortier L-C, Bikard D, Monot M. 2017. PhageTerm: a tool for fast and accurate determination of phage termini and packaging mechanism using next-generation sequencing data. Sci Rep 7:8292. doi:10.1038/s41598-017-07910-5. 28811656 PMC5557969

[7] Laslett D, Canback B. 2004. ARAGORN, a program to detect tRNA genes and tmRNA genes in nucleotide sequences. Nucleic Acids Res 32:11–16. doi:10.1093/nar/gkh152. 14704338 PMC373265

[8] Besemer J, Lomsadze A, Borodovsky M. 2001. GeneMarkS: a self-training method for prediction of gene starts in microbial genomes. implications for finding sequence motifs in regulatory regions. Nucleic Acids Res 29:2607–2618. doi:10.1093/nar/29.12.2607. 11410670 PMC55746

[9] Delcher A. 1999. Improved microbial gene identification with GLIMMER. Nucleic Acids Res 27:4636–4641. doi:10.1093/nar/27.23.4636. 10556321 PMC148753

[10] Seemann T. 2014. Prokka: rapid prokaryotic genome annotation. Bioinformatics 30:2068–2069. doi:10.1093/bioinformatics/btu153. 24642063

[11] Altschul SF, Gish W, Miller W, Myers EW, Lipman DJ. 1990. Basic local alignment search tool. J Mol Biol 215:403–410. doi:10.1016/S0022-2836(05)80360-2. 2231712

[12] Aiewsakun P, Adriaenssens EM, Lavigne R, Kropinski AM, Simmonds P. 2018. Evaluation of the genomic diversity of viruses infecting bacteria, archaea and eukaryotes using a common bioinformatic platform: steps towards a unified taxonomy. J Gen Virol 99:1331–1343. doi:10.1099/jgv.0.001110. 30016225 PMC6230767

